# Low-cost wound protector for laparoscopic surgeries

**DOI:** 10.1590/0100-6991e-20243765-en

**Published:** 2024-07-05

**Authors:** CARLOS AUGUSTO METIDIERI MENEGOZZO, CRISTIANE PRESTES DA ROSA OLIVEIRA, ROBERTO RASSLAN, FELIPE KFOURI, ALBERTO BITRAN, RENATO SILVEIRA LEAL, SERGIO HENRIQUE BASTOS DAMOUS, EDIVALDO MASSAZO UTIYAMA

**Affiliations:** 1 - Universidade de São Paulo, Disciplina de Cirurgia Geral e Trauma - São Paulo - SP - Brasil.

**Keywords:** Wound Infection, Surgical Wound Infection, Hand-Assisted Laparoscopy, Laparoscopy, Infecção da Ferida Cirúrgica, Ferida Cirúrgica, laparoscopia, Laparoscopia Assistida com a Mão

## Abstract

The role of wound protectors in laparoscopic surgeries is highly controversial in the literature. Some studies demonstrate their benefit in reducing the rate of surgical site infections; however, these results are not reproducible across all procedures. In addition to protecting the operative wound, these devices can be used at sites of surgical specimen extraction in laparoscopic procedures. Several commercially available devices serve this purpose but are scarcely available in resource-limited settings. One of the reasons for this limitation is the cost of the device. In this technical note, we aim to provide a cost-effective option utilizing materials readily available in the operating room and with a simple fabrication process.

## RESUMO

O papel dos protetores de ferida operatória em cirurgias laparoscópicas é bastante controverso na literatura. Alguns estudos demonstram seu benefício na redução da taxa de infeções de sítio cirúrgico, porém esses resultados não são reprodutíveis em todos os procedimentos. Além da proteção da ferida operatória, esses dispositivos podem ser utilizados nos sítios de extração de peças cirúrgicas em procedimentos laparoscópicos. Há vários dispositivos comercialmente disponíveis para esse fim, entretanto são pouco disponíveis nos serviços com menos recursos. Um dos motivos dessa limitação é o custo do dispositivo. Nesta nota, buscamos oferecer uma opção barata que utiliza materiais amplamente disponíveis no centro cirúrgico e cuja confecção é simples.

## INTRODUCTION

Surgical site infection (SSI) is a frequent situation, associated with higher morbidity and mortality^1^. The incidence of SSI is estimated to be 2% to 5% in surgeries in hospitalized patients^2^. In addition to the increase in morbidity, SSI results in longer hospital stays and higher hospital costs^1^.

The use of wound protectors is one of the proposals to mitigate the risk of SSI^3^. Studies show that these devices can reduce the risk of SSI in conventional surgeries, but the evidence for laparoscopic surgeries is less robust^4^ ^-^ ^7^. Despite the potential benefit, the cost of commercial surgical wound protectors limits their use, and they are poorly available in services with fewer resources. In these conditions, the surgeon may use sterile materials for surgical wound protection to decrease infection rates. However, adapting materials and making devices that simulate commercial products can be difficult.

The main objective of this Technical Note is to describe a low-cost surgical wound protector option that mimics the functioning of commercial protectors in a satisfactory way.

## TECHNIQUE DESCRIPTION

The manufacture of the surgical wound protector requires a number 8.0 sterile surgical glove, a semi-rigid plastic tube, and a 2-0 Nylon thread, totaling a cost of R$ 4.87 in our institution (around US$ 0.92). It can be made by the scrub nurse during the surgical procedure, avoiding an increase in surgical time. The description of the step-by-step process for making the device is shown in Table 1.

**Table 1:** Ten steps to making a low-cost surgical wound protector.

Stage	Description	Figure
Step 1	Division of the ends of the suction tube	1a
Step 2	Suction tube cross-section in 4 equal parts	1c
Step 3	Making of a 2-cm "slit" at one end of two of the parts of Step 2	1d
Step 4	Division of the entire length of the remaining two parts of Step 2	
Step 5	Creation of the inner halo with the two parts obtained in Step 3	2a
Step 6	Positioning of the proximal portion of the sterile glove in the inner halo	2b
Step 7	Positioning of the external halo over the proximal portion of the sterile glove	2c
Step 8	Division of the distal portion of the sterile glove	3a
Step 9	Repeat Steps 6 and 7 at the distal end of the sterile glove	3b
Step 10	Cardinal fixation sutures	3d

**Figure 1:**
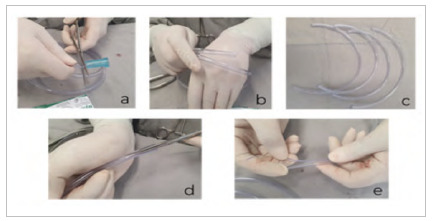
Steps for making the internal and external halos that will serve as the ends of the surgical wound protector.

The first step consists of preparing the sterile semi-rigid plastic tube. The suction tube is used with the extremities (connections) divided with scissors (Figure 1a). Next, the plastic tube is divided into four equal segments, whose lengths are 1 to 2 cm greater than the wrist perimeter of the sterile surgical glove (Figures 1b and 1c). Two of these segments will be used to construct the internal halo to support the wound protector and, for this, a section of approximately 2 cm is performed at one end of each separate segment (Figure 1d and 1e).

**Figure 2:**
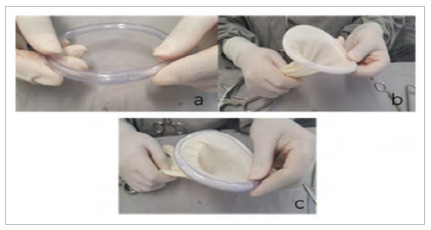
Steps for making one of the ends of the surgical wound protector consisting of the sectioned sterile glove and two halos (internal and external).

The two remaining segments will be used to make the external support halo and, for this, a longitudinal section similar to Figure 1d will be performed, but from one end to the other, along the entire length of the two remaining plastic tube segments. Next, the divided end is positioned so that it surrounds the intact end, creating a circle (inner halo) (Figure 2a). 

Next, the inner halo is covered by the proximal part of the sterile glove (Figure 2b), and one of the external halos is positioned on top of it, to keep the proximal segment of the sterile glove fixed between the inner and outer halos (Figure 2c). 

**Figure 3:**
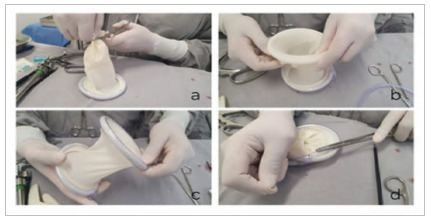
Steps for making the other end of the surgical wound protector and attaching the halos to the sterile glove.

The next step is to cut the distal portion of the sterile glove (which contains the “glove fingers”) to keep only the wrist segment of the glove (Figure 3a). The diameter of the glove should be equal to or greater than the length of the incision in the aponeurosis, through which the surgical specimen will be removed. This ensures a more suitable seal by the elastic fabric of the sterile glove. Then, the second pair of plastic halos is used to wrap around the distal end of the sectioned sterile glove (Figure 3b). The product is a cylinder whose two ends are open, connected by the “handle” of the sterile sleeve (Figure 3c).

Finally, four cardinal 2-0 nylon sutures sutures are performed, transfixing the internal and external halos of both ends, providing more stability to the entire set (Figure 3d).

## DISCUSSION

This technical note presents a low-cost, easy-to-make option for the protection of small surgical wounds. It is a technique that requires common materials, with the potential to offer benefits in reducing the incidence of surgical wound complications.

SSI is an important public health issue. According to the Centers for Disease Control and Prevention (CDC), around 20% of nosocomial infections are surgical and they increase the risk of death by two to 11 times, as well as the length of hospital stay and hospital costs^1^.

The incidence of SSI varies according to several perioperative factors. Among them, the operated region, the size of the incision, the patient’s comorbidities, and whether the procedure is elective or urgent^3^. Considering the type of surgery, the incidence of surgical wound infection ranges from 2.6%-5%, 6.7%-11%, 8.6%-17%, and 12%-27% in clean, potentially contaminated, contaminated, and infected surgeries, respectively^8^. The presence of active infection at the incision site can result in SSI in up to 40% of cases and can reach 50% in critically ill patients9. While some factors are not modifiable, some perioperative strategies can be adopted to mitigate the risks of SSI. Among them, surgical wound protectors present good results, especially in contaminated sites^9^.

Abdominal surgery is considered one of the most prone to SSI, with rates between 15% and 25%. The surgical access route itself also influences the incidence of SSI. According to Alkaaki et al., minimally invasive surgery has lower SSI rates when compared with laparotomy, being 0.02% in clean surgeries, 3.2% in potentially contaminated surgeries, and 17% in infected ones^10^. In this study, the overall rate of SSI was approximately ten times lower in laparoscopic surgeries. 

The use of surgical wound protectors in abdominal surgery has controversial results in the literature, but there seems to be greater benefit in colorectal and bile duct surgeries^3^ ^,^ ^11^. A study with 625 patients who underwent colorectal surgery via laparotomy demonstrated that the use of protectors resulted in a lower incidence of surgical wound infection (3.2% vs 11.2%) and SSI (8% vs 13.7%), with statistical significance^5^. Li et al. published a meta-analysis with more than 4,000 patients and confirmed the benefit of surgical wound protectors in various types of abdominal surgery but emphasized that this benefit was not observed in colorectal surgeries^4^. Meta-analyses evaluating the benefit of incision protectors in appendectomies and pancreaticoduodenectomies also suggest a benefit in reducing the incidence of SSIs^7^ ^,^ ^12^.

Most of the published studies analyze procedures performed by laparotomy, while studies on the use of surgical wound protectors in laparoscopic surgery are scarce and have a lower level of evidence. Kercher et al. retrospectively analyzed 141 patients who underwent video-assisted colectomy and did not identify benefits of the use of surgical wound protectors in terms of SSI incidence (12% vs 14% in the groups with and without surgical wound protector, respectively)^6^. Luo et al. published a study with a similar design including 109 patients with different outcomes, in which the group using surgical wound protectors had a lower incidence of SSI (1.7% vs 13.4%) and shorter mean length of hospital stay (7 days vs 8 days). These studies are limited by their retrospective nature, with risk of bias, and by the variability of perioperative practices that may impact the occurrence of SSI. 

A similar device has been previously described for the removal of surgical specimens from laparoscopic colectomy due to colon neoplasia and endometriosis^13^. The authors demonstrate a device using a 20Fr urethral catheter and the sterile polyethylene plastic used to protect the fiber optic cable. The removal of the specimen and the maintenance of the pneumoperitoneum are done by manipulating two Kelly forceps. The authors showed good results with the use of this device in six patients. Regarding that device, this technical note presents an option with a similar objective, low cost, and with an elastic lumen. This technical note does not include an analysis of manufacturing parameters or postoperative results. However, considering the mean time reported by the authors to make the device with a plastic cover of 66 seconds^13^, it is safe to report that what is described in this technical note requires more time for its manufacture. The decision to use the plastic device with the sterile glove was due to the apparent ease of handling the device, which was used satisfactorily in three cases of laparoscopic rectosigmoidectomy.

**Figure 4:**
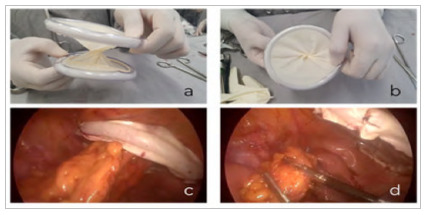
Twisting maneuver of the surgical wound protector that allows the maintenance of the pneumoperitoneum in laparoscopic surgeries, outside the operative field (a and b), and in the operative field, after the removal of the specimen (c), demonstrating the device’s ability to maintain an adequate pneumoperitoneum (d).

In addition to the potential benefit of reducing the incidence of SSI, commercial elastic devices allow separation of the incision edges by increasing the tension on the plastic, and a temporary closure of the incision by “twisting” the plastic of the device, avoiding the loss of pneumoperitoneum during laparoscopy. In the case of the device described in this article, the fragility of the sterile glove limits the ability to move the edges of the incision away by increasing the plastic tension. However, it is possible to perform a satisfactory temporary closure of the incision by twisting the device (Figures 4a and 4b). Thus, after removal of the specimen (Figure 4c), it is possible to maintain the pneumoperitoneum adequately, allowing the continuation of the procedure (4d). The device described in this article can be used on any surgical wound if the size of the incision in the wound greatly exceeds the diameter of the sterile glove used. Also, it can potentially be applied for extra-abdominal surgeries.

## CONCLUSION

This article offers a technical description of the manufacture of a low-cost and easy-to-perform surgical wound protector, with the potential to reduce surgical wound complications, even in services that do not have the resources to purchase the commercial device.
